# Polyphenols and Antioxidant Activity of Citrus Fiber/Blackberry Juice Complexes

**DOI:** 10.3390/molecules26154400

**Published:** 2021-07-21

**Authors:** Ivana Buljeta, Anita Pichler, Josip Šimunović, Mirela Kopjar

**Affiliations:** 1 Faculty of Food Technology Osijek, Josip Juraj Strossmayer University of Osijek, F. Kuhača 18, 31000 Osijek, Croatia; ivana.buljeta@ptfos.hr (I.B.); anita.pichler@ptfos.hr (A.P.); 2 Department of Food, Bioprocessing and Nutrition Sciences, North Carolina State University, Raleigh, NC 27695, USA; simun@ncsu.edu

**Keywords:** citrus fibers, blackberry juice, antioxidants, polyphenols profile

## Abstract

The objective of this study was to investigate the use of citrus fiber as a carrier of blackberry juice polyphenols. For that purpose, freeze-dried complexes with blackberry juice and different amounts of citrus fiber (1%, 2% and 4%) were prepared. Complexes were evaluated spectrophotometrically for total polyphenols, proanthocyanidins and antioxidant activity. Analyses of individual polyphenols were performed using high-performance liquid chromatography. IR spectra were recorded to confirm encapsulation. All analyses were performed after preparation and after eight months of storage, in order to examine the stability of formed complexes. The obtained results indicated that increasing the amount of fiber led to a decrease in the concentration of polyphenols and the antioxidant activity of complexes. Cyanidin 3-glucoside was the prevalent anthocyanin in complexes (138.32–246.45 mg/100 g), while cyanidin 3-dioxalylglucoside was present at lower concentrations (22.19–31.45 mg/100 g). The other identified and quantified polyphenols were hesperidin (from citrus fiber), ellagic acid and quercetin (1317.59–1571.65 mg/100 g, 31.94–50.11 mg/100 g and 20.11–33.77 mg/100 g, respectively). Degradation of polyphenols occurred during storage. Results obtained in this study confirmed that citrus fiber could be used for the formulation of novel bioactive additives. Such additives could enhance the antioxidant potential of products to which they are added, such as baked goods, dairy, or fruit products.

## 1. Introduction

The modern food industry is increasingly utilizing food waste streams (upcycling), and citrus waste is of increasing interest since it offers a wide range of possible applications. Citrus fiber, obtained by extraction from citrus peel (mandarin, orange, lemon, and lime) is a natural dietary fiber [1]. Pectin makes up the bulk of citrus fiber, but hemicellulose and cellulose are also present [2]. The binding capacity, apparent viscosity, water-holding capacity and internal surface area of citrus fiber are higher than in some other fiber, such as carrot or oat fibers, leading to its wider application range in various products in the food industry (dairy, baked goods, meats, dressings, and sauces) [2]. Many positive properties related to maintaining health and reducing the risk of various diseases (hypertension, type 2 diabetes, obesity, etc.) [3] have been attributed to dietary fiber, and more emphasis is now placed on the consumption of fiber-rich products. Food products enriched with fibers from various sources are therefore becoming increasingly popular.

Blackberries contain numerous bioactive compounds. In addition to polyphenols, which are of great interest for the food and nutraceutical industries, the presence of other components, such as fibers, vitamins, minerals and sugars, is also significant [4,5]. Polyphenols are naturally occurring compounds formed as secondary metabolites in plants, which, due to their structural complexity, are classified into phenolic acids, flavonoids, tannins, stilbenes, coumarins and polymeric ligands [6]. In addition to cyanidin 3-glucoside, which is the most abundant anthocyanin in blackberries [7], many phenolic acids, tannins and other anthocyanins are also present, contributing to the high antioxidant capacity of these plants [8,9]. The chemical composition of blackberries and the content of polyphenols depend on the varieties, growing conditions, location, stage of maturity and harvest [5]. Interest in the healing properties of blackberries dates back to the sixteenth century in Europe, where they were used to relieve infections of the eyes and mouth [10]. Consumption of blackberries, as well as other products rich in polyphenols, was associated with the prevention of cardiovascular diseases [11] and antimicrobial, anticancer, antimutagenic, neuroprotective, antiproliferative and anti-inflammatory activity [5,10,12]. In addition to the consumption of fresh blackberries, blackberries are used to make many products, such as jams, juices, purees, sauces, cakes, ice cream, pies/cobblers, marmalade, wine, liqueurs, canned and dried blackberries [13,14].

The enrichment of conventional products and the development of novel foods, which meet demands for healthier foods, is currently a challenge for the food industry [15,16]. In order to meet those demands, bioactive blackberry compounds could be used as a source of valuable natural additives [17].

Choosing an encapsulation method, as well as developing a carrier for valuable components that protects them but at the same time is inexpensive and safe for consumption, is a demanding process [16]. Polysaccharides such as starch, pectin, cellulose, and β-cyclodextrin have been recognized as some of the potentially successful materials for encapsulation [18,19,20,21]. Dietary fibers and polyphenols have usually been studied separately, but it is necessary to understand their interactions since this can affect the bioavailability and bioaccessibility of polyphenols that have been examined in numerous studies [14,16,22,23,24,25,26]. In addition, there is evidence that different fibers (such as strawberry dietary fibers, apple cell wall components, and onion fiber) can be utilized to deliver different polyphenols and to be used for the efficient preparation of functional food ingredients [27,28,29,30].

The encapsulation of bioactive compounds such as polyphenols is an active research field, and the main goals are to overcome their low storage stability and obtain a suitable protection system [16]. Unsaturated bonds in the polyphenols’ structure make them sensitive to oxidizing agents (light, heat, moisture and oxygen) and exposure to those agents could cause structural changes. Freeze-drying is a suitable drying technique for heat-sensitive compounds such as polyphenols, since the temperature levels used in this process are low [31].

The objective of this study was to prepare freeze-dried citrus fiber/blackberry juice complexes and evaluate the influence of different amounts of fiber on the polyphenol profile and antioxidant activity. In addition, FTIR analysis was carried out to confirm the adsorption of polyphenols onto the carrier. Formed complexes were stored for eight months to examine their stability under given conditions (light and room temperature). To the best of our knowledge, this is the first study conducted on citrus fiber and blackberry juice freeze-dried complexes. These complexes could be utilized as novel bioactive ingredients in various food products (such as baked goods, dairy, fruit products, etc.) in order to improve their properties. In addition, these novel additives could be used in the domestic preparation of smoothies and shakes, which are quite popular products from the consumers’ perspective, in order to enrich these foodstuffs with fibers and phenolics.

## 2. Results and Discussion

### 2.1. Water Activity (a_w_) of Freeze-Dried Complexes

Water activity refers to the available water for biological functions. By reducing water activity, the growth ability of most microorganisms decreases. The limit value of a_w_ for the growth of any microorganism is about 0.6, and below this value, food is considered microbiologically safe. Table 1 shows the values of a_w_ for the complexes after preparation and after storage.

### 2.2. Polyphenols of Citrus Fiber/Blackberry Juice Complexes

The blackberry juice used in this study was analyzed for total polyphenols, proanthocyanidins, and individual polyphenols, as well as antioxidant activity. The characteristics of blackberry juice are presented in Table 2.

In Table 3, the total polyphenol and proanthocyanidin contents in citrus fiber/blackberry juice complexes, after preparation and after storage (eight months), are presented. The complex with 1% of fiber had the highest levels of polyphenol content after preparation (12.89 mg/g) and, as the percentage of citrus fiber increased (2% and 4%), the amounts of total polyphenol content in complexes decreased (7.49 mg/g and 6.27 mg/g, respectively). A similar downward trend with an increasing citrus fiber percentage was observed for proanthocyanidins and ranged from 1.15 to 1.90 mg/g. The proanthocyanidin content was the highest for the complex that had 1% of fiber (1.90 mg/g).

During the eight months of storage, the degradation of polyphenols occurred, and the stored complexes had lowered post-storage total polyphenol and proanthocyanidin contents. This trend, observed after preparation, also remained after storage, i.e., with an increase in the amount of fiber, a decrease in polyphenols was observed. However, by calculating the retention of polyphenols after storage, it was observed that complexes with higher amounts of fibers had higher retention levels of polyphenols. For total polyphenols, retention was 50%, 70% and 79% for those complexes with 1%, 2% and 4% of fiber, respectively, while for proanthocyanidins, retention was 74% for complexes with 1% and 2% of fiber, and 80% for the complex with 4% of fiber.

Individual polyphenols present in the complexes were determined by HPLC analyses. Those polyphenols identified and quantified in obtained complexes are shown in Table 4. In blackberry juice, cyanidin 3-glucoside, cyanidin 3-dioxalylglucoside, ellagic acid, p-coumaric acid, caffeic acid, chlorogenic acid, gallic acid, quercetin and rutin were determined (Table 2). The identified compounds were in accordance with the values found in the literature [32]. Regarding complexes, cyanidin 3-glucoside, cyanidin 3-dioxalylglucoside, ellagic acid, quercetin and hesperidin were detected. The presence of hesperidin in citrus fruits resulted in its presence in the citrus fiber, and it was consequently identified in the complexes. Hesperidin is considered one of the main flavonoids in citrus fruits and has been attributed positive health benefits [33]. Polyphenols from various plant sources can be adsorbed onto dietary fibers and/or proteins through hydrogen interactions, hydrophobic bonds, and/or ether and ester covalent bonds in plant material [34], which could have caused the appearance of hesperidin in our complexes. The presence of other polyphenols in complexes was from blackberry juice. Cyanidin 3-glucoside was the prevalent anthocyanin, and its concentration in complexes before storage was 246.45 mg/100 g, 243.57 mg/100 g, and 160.78 mg/100 g for complexes with 1%, 2%, and 4% of fiber, respectively. Furthermore, cyanidin 3-dioxalylglucoside was present in lower concentrations (31.45 mg/100 g, 22.67 mg/100 g and 20.51 mg/100 g for 1%, 2% and 4% of citrus fiber), following the downward trend with increasing amounts of fiber. In addition to anthocyanins and hesperidin (1571.65 mg/100 g, 1422.25 mg/100 g and 1371.88 mg/100 g for complexes with 1%, 2% and 4% of fiber, respectively), the other identified and quantified polyphenols in the complexes were ellagic acid and quercetin, and in the complexes before storage, their concentrations ranged from 22.96 mg/100 g to 50.11 mg/100 g, and from 20.43 mg/100 g to 33.77 mg/100 g, respectively. It was observed that the trend remained the same: with the increasing fiber amount, the adsorption of polyphenols decreased. Finally, it can be concluded, regarding the complexes after preparation, that the highest concentration of all polyphenols was in the complex with 1% of fiber.

Cyanidin 3-glucoside is the dominant anthocyanin in blackberries at a ripened stage, while in the intermediate stage it is cyanidin 3-dioxalylglucoside [35]. Ellagic acid belongs to a group of hydroxybenzoic acids and is one of the main polyphenols in blackberries [35]. Polyphenols and dietary fibers are able to create chemical interactions in foods [36]. These interactions can occur across non-covalent bonds, such as hydrogen bonds (between oxygen atoms of the glycosidic bonds of polysaccharides and hydroxyl groups of polyphenols), hydrophobic interactions and van der Waals forces [22]. In a study by Phan et al. [37], molecular interactions between cellulose and selected polyphenols (gallic acid, chlorogenic acid, ferulic acid, cyanidin-3-glucoside and (+/-) catechin) were studied. The interactions of polyphenols and cellulose appeared within 1 min of the initial contact, and increased within 30 min, while after 2 h there was no significant increase. Furthermore, higher molecular weight polyphenols have been shown to bind more to cellulose than lower molecular weight ones. This observation was consistent with the present study, where phenolic acids from blackberry juice (chlorogenic, caffeic, gallic and p-coumaric acids) were not determined in complexes, due to their lower molecular weights in comparison with anthocyanins, which have a higher molecular weight. The bonds between polyphenols and cellulose (hydrogen bonds and hydrophobic interactions) largely depend on the number of aromatic rings and hydroxyl groups in the polyphenol structure [38]. In addition, a secondary factor is the native charge of phenolic compounds that can be seen in the example of the lower maximum adsorption capacity of phenolic acids (gallic acid, chlorogenic acid, ferulic acid) that are bound to cellulose. It is considered that there is a rejection between the electronegative hydroxyl group of cellulose and the negatively charged phenolic acids [37]. The binding of phenolic acids and anthocyanins to cellulose and cellulose-pectin (a system for the simulation of purple carrot (*Daucus carota*) cell wall) showed a higher affinity for cellulose, and it is assumed that the supramolecular arrangement of the carrier affected the binding [39]. According to the research conducted by Zhang et al. [40], hydrogen bonds and weak hydrophobic interactions have been proven between ferulic acid and arabinan-rich pectic polysaccharides. Fernandes et al. [41] used low methoxylated pectin (14%) from citrus fruits to study the interactions between anthocyanins (cyanidin 3-glucoside and delphinidin 3-glucoside) and pectin. Using UV-Vis and saturation transfer difference nuclear magnetic resonance (STD-NMR) spectroscopy, weak interactions between pectin and anthocyanins were observed. Hydrogen bonds and hydrophobic interactions have been hypothesized as being responsible for these interactions. Higher binding with pectin was observed for delphinidin 3-glucoside, which contains a pyrogallol moiety with three hydroxyl groups in its structure, more than for cyanidin 3-glucoside with a catechol moiety (two groups); this indicates that the number of hydroxyl groups in the anthocyanin ring affected the binding. We can also conclude that hydrogen bonds and hydrophobic interactions were involved in the binding of blackberry juice polyphenols onto citrus fiber. The amount of bonded blackberry juice polyphenols onto the citrus fiber definitely depends on the number of available binding sites. We cannot neglect the fact that, during complexation, citrus fibers can interact not only with polyphenols but also among themselves, lowering the number of binding sites available for polyphenols. In addition, the stability of hydrogen bonds and hydrophobic interactions over time were responsible for the stability of complexes, i.e., stability of phenolic compounds over the storage period.

In addition, it can be assumed that the adsorption of polyphenols onto fiber was concentration-dependent [26]. In a study by Jakobek et al. [26] and Phan et al. [37], polyphenols that were present in higher concentrations in the fiber environment were adsorbed in higher amounts, which agrees with our results, where cyanidin 3-glucoside was in high amounts in blackberry juice, and consequently in the complexes. The concentration of hesperidin (whose presence originated from the citrus fiber) on the complexes decreased with the increasing amount of fiber, and it could be hypothesized that there was a competition with other polyphenols for binding sites on the fiber. Furthermore, it was observed that with the further increase in fiber amounts, there was no increase in the adsorption of polyphenols, which could mean that at one point the maximum binding capacity was achieved, more precisely, on the complex with 1% of fiber. Future experiments could be performed on intermediate percentages, in order to confirm and optimize these findings.

During storage, a change to polyphenols occurred. The concentration of cyanidin 3-glucoside in complexes with 1% and 2% of fibers was constant at 214 mg/100 g, while for the complexes with 4% of fibers, it was 138.32 mg/100 g. The same trend was observed for cyanidin 3-dioxalylglucoside; thus, complexes with 1% and 2% of fibers had 25.6 mg/100 g of this anthocyanin, and the complex with 4% of fiber, 22.19 mg/100 g. The concentrations of ellagic acid, quercetin and hesperidin ranged from 21.94 mg/100 to g 44.55 mg/100 g, from 20.11 mg/100 g to 24.75 mg/100 g, and from 1317.59 mg/100 g to 1358.84 mg/100 g, respectively, with an identical effect observed for different fiber amounts as for the complexes before storage, i.e., with the increase in fiber amount, a decrease in phenolic content was observed. Even though the concentrations of polyphenols were highest for the complex with 1% of fiber, a comparison of retentions indicated that higher percentage retention of anthocyanins, ellagic acid, quercetin and hesperidin was achieved for complexes with 2% and 4% of fibers. The degradation of anthocyanins was more pronounced than for other phenolic compounds. A study by Murali et al. [42] showed that prolonged storage leads to the greater degradation of anthocyanins in freeze- and spray-dried powders of black carrots with combined carrier materials (maltodextrin, gum arabic, tapioca starch).

### 2.3. Antioxidant Activity of Citrus Fiber/Blackberry Juice Complexes

Blackberry juice is rich in antioxidants and, therefore, it was necessary to determine the antioxidant capacity of our complexes. Many methods for measuring antioxidant activity have been given in the literature, but there is no method that shows the mechanisms of action of all antioxidants present in a complex system such as food. For this reason, multiple methods are often combined to obtain an antioxidant potential of all present antioxidants. In this study, the antioxidant activities of complexes were estimated by the application of four assays, and the results are presented in Table 5. The DPPH method is based on free radical scavenging activity, and its broad application is the result of its simplicity and speed, and its inexpensive equipment [43]. Due to their better access to the reactive site, small molecules show high antioxidant activity, as determined by the DPPH assay. For complexes after preparation, the results of the DPPH assay ranged from 36.48 µmol/100 g to 50.39 µmol/100 g. The complex with 1% fiber had the highest level, while an increased amount of fiber caused a decrease in antioxidant activity. The results of the ABTS assay followed a similar trend and ranged from 29.60 µmol/100 g to 52.83 µmol/100 g. CUPRAC and FRAP assays were used for evaluation of the ability of antioxidants to reduce cupric and ferric ions, respectively.

The obtained results for FRAP and CUPRAC assays ranged from 6.65 µmol/100 g to 9.71 µmol/100 g, and from 362.00 µmol/100 g to 515 µmol/100 g, respectively, with the previously observed trend of decreasing antioxidant activity with the increasing amounts of fiber. For results obtained by the DPPH, CUPRAC and FRAP assays, it was observed that there was no significant difference in antioxidant activity between the complexes with 1% and 2% of fiber. Our results are in agreement with a study by Vukoja et al. [21], where an increase in the amount of cellulose in the freeze-dried complexes of cellulose and raspberry polyphenols led to a decrease in antioxidant activity.

The antioxidant activity of stored complexes, measured with four different assays, was lower in comparison with freshly prepared complexes, following the same trend. Results for the DPPH, ABTS, FRAP and CUPRAC assays ranged as follows: 35.48–49.84 µmol/100 g, 24.78–51.30 µmol/100 g, 6.30–9.13 µmol/100 g and 351.34–509.41 µmol/100 g, respectively. Herein, the decrease of the antioxidant potential of complexes corresponded to the polyphenol concentrations determined by spectrophotometric and HPLC analysis. 

### 2.4. IR Spectra of Citrus Fiber/Blackberry Juice Complexes

**Figure 1:**
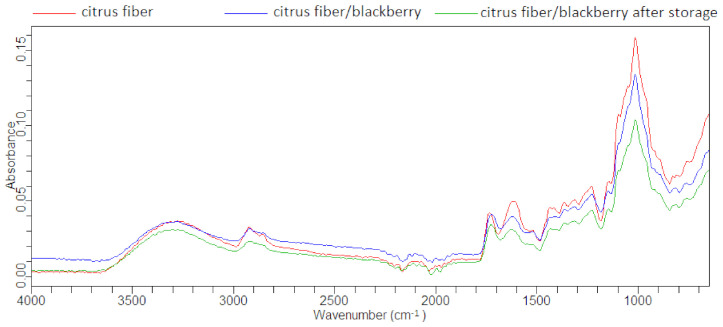
IR spectra of citrus fiber and citrus fiber/blackberry juice complexes after preparation and after storage.

The FTIR technique was used to observe not only the main functional groups present in citrus fiber and obtained complexes, but also to confirm that blackberry juice polyphenols were adsorbed and penetrated the interlayer space of the citrus fiber. Figure 1 presents the IR spectra of citrus fiber in comparison with citrus fiber/blackberry juice complexes after preparation and after storage. The spectrum of one complex was chosen for comparison because the others had identical differences. The major differences in the IR spectra of citrus fiber and complexes were observed in the regions 3000–2800 cm^−1^ and 1700–700 cm^−1^. The overlapping spectra of citrus fiber and the complex band at 3280 cm^−1^ could be assigned to the O-H stretching vibration. Generally, the region between 3200 and 3600 cm^−1^ is assigned to free and intermolecular bound hydroxyl groups that are associated with many O-H groups on pectin. The bands at 2922 cm^−1^ and 2856 cm^−1^ could be related to the C-H stretching vibration [44]. It is also important to notice that the intensities of these two bands decreased in the complexes. The band at 1735 cm^−1^ indicated the presence of the C=O stretching vibration of the alkyl ester of polysaccharides, such as pectin and hemicellulose. Furthermore, the band at 1610 cm^−1^ can be assigned to COO^-^ antisymmetric stretching, which can be related to polygalacturonic acid and carboxylate (pectin ester group), while the 1438 cm^−1^, 1364 cm^−1^ and 1312 cm^−1^ bands can be assigned to CH_2_ symmetric bending in cellulose. The band at 1230 cm^−1^ is connected to C-O stretching, and band at 1013 cm^−1^ besides C-O includes C-C stretching in the pectin ring structure [45].

The band at 829 cm^−1^ can be assigned to the *C*_2_’-endo conformation of sugar, and that band was lost or had very low intensities in complexes, while the band around 813 cm^−1^ can be assigned to ring C-H deformation, and all bands between 600 cm^−1^ and 900 cm^−1^ can be assigned to C-H out-of-plane bending vibrations. In addition, it was observed that the band intensities (in the region between 600 and 1700 cm^−1^) decreased in the IR spectrum when blackberry juice polyphenols were loaded, which was in agreement with a study by Abdelwahab and Amin [46], where, when the adsorption of polyphenols onto *Luffa cylindrica* fibers were investigated, a decrease in band intensities was observed.

## 3. Materials and Methods

### 3.1. Materials

Citrus fiber (76.1% of dietary fiber, while the rest are proteins—7%, sugars—7.2%, fats—0.77%, minerals—3.06%, and moisture—5.85%) was obtained from Fiberstar (River Falls, WI, USA). Potassium persulfate and sodium carbonate were purchased from Kemika (Zagreb, Croatia). Neocuproine, hesperidin, 2,4,6-tri(2-pyridyl)-s-triazine (TPTZ) and copper (II) chloride were bought from Acros Organics (Geel, Belgium). Trolox, 2,2-diphenyl-1-picrylhydrazyl (DPPH), 2,2′-azino-bis(3-ethylbenzothiazoline-6-sulfonic acid) diammonium salt (ABTS), 4-(dimethylamino)-cinnamaldehyde (DMAC), quercetin, caffeic acid, p-coumaric acid, chlorogenic acid, rutin, gallic acid and ellagic acid were obtained from Sigma-Aldrich (St. Louis, MO, USA). Cyanidin 3-glucoside was from Extrasynthese (Genay, France). Methanol (HPLC grade) was from J.T. Baker (Deventer, Netherlands), and orthophosphoric acid (HPLC grade > 85%) was from Fisher Scientific (Loughborough, UK). Iron (III) chloride hexahydrate, sodium acetate, ethanol, ammonium acetate and Folin–Ciocalteu reagent were bought from Gram-mol (Zagreb, Croatia).

### 3.2. Preparation of Bioactive Food Complexes 

Blackberry juice was obtained by pressing blackberry fruit through a cheesecloth. The preparation of citrus fiber/blackberry juice complexes was carried out by mixing blackberry juice (50 mL) and citrus fiber (1%, 2% and 4%) using a magnetic stirrer (Stuart US152, Buch and Holm, Hervel, Denmark) for 15 min at room temperature. Thereafter, the obtained mixtures were centrifuged for 15 min at 4000 rpm (Microspin 12, Grant Instruments Ltd, Royston, UK) to separate the liquid part from the precipitate. To obtain a dry powder, the precipitate was freeze-dried. Prior to freeze-drying in the laboratory freeze-dryer (Christ Freeze Dryer, Alpha 1-4, Osterode am Harz, Germany), the precipitate was frozen for 24 h at −18 °C. Freeze-drying took place under the following conditions: the freezing temperature was adjusted to −55 °C; the temperature of sublimation ranged from −35 °C to 0 °C; and the vacuum level was at 0.220 mbar. The temperature of the isothermal desorption varied from 0 °C to 21 °C under a vacuum of 0.060 mbar. The complete process lasted for 12 h. The obtained dry complexes were divided into two parts, one part of which was stored for eight months at room temperature and exposed to light, while the other part was analyzed immediately after preparation. The complexes were stored in transparent glass jars with metal lids. The same analyses were performed with both freshly prepared and stored samples.

### 3.3. Determination of Water Activity (a_w_) in Freeze-Dried Complexes

The water activity of the freeze-dried complexes was determined using a water activity meter (METER Group, Inc., Pullman, WA, USA). Prior to measurements, the device was calibrated using the standards LiCl (13.41 M) and NaCl (6 M) with a_w_ values of 0.247 and 0.760, respectively.

### 3.4. Extraction of Polyphenols

Approximately 0.3 g of the dry complex was extracted with 15 mL of acidified methanol (the methanol:HCl ratio was 99:1). The extraction was carried out at room temperature for 24 h, followed by filtration. The extracts were subjected to an HPLC evaluation of polyphenols and a spectrophotometric determination of total polyphenols, proanthocyanidins and antioxidant activity.

### 3.5. Determination of Total Polyphenols and Proanthocyanidins Contents

#### 3.5.1. Total Polyphenol Content

Total polyphenol content was determined through a modified colorimetric Folin–Ciocalteu method [47]. An aliquot (0.2 mL) of the extract was mixed with 1.8 mL of deionized water, followed by the addition of 10 mL (1:10) Folin–Ciocalteu reagent and 8 mL of sodium carbonate solution (7.5%). After 120 min at room temperature, when the color had developed, the absorbance was read at 765 nm on a UV/Vis spectrophotometer (Cary 60 UV-Vis, Agilent Technologies, Santa Clara, CA, USA). The calibration curve for gallic acid was construed in the concentration range from 0.5 to 4 mg/mL, and results were expressed as mg of gallic acid equivalents per g of sample (mg GAE/g).

#### 3.5.2. Proanthocyanidin Content

The proanthocyanidin content was determined by the DMAC method [48]. The absorbance of samples was measured at 640 nm, and the results were expressed as mg of procyanidin B2 equivalent per g of sample (mg PB2E/g). The calibration curve for procyanidin B2 was construed in the concentration range from 6 to 400 µg/mL.

### 3.6. High-Performance Liquid Chromatography (HPLC) Evaluation of Polyphenols

Polyphenols in the extracts of complexes, as well as in blackberry juice and citrus fiber extract, were analyzed using an Agilent HPLC system 1260 Infinity II (Agilent Technology, Santa Clara, CA, USA) according to the method described by Ivić et al. [49]. Prior to injection into the system, 1 mL of extract was filtered through a 0.20 µm PTFE syringe filter. The system consisted of a quaternary pump, a diode array detector (DAD), a vial sampler and a Poroshell 120 EC C-18 column (4.6 × 100 mm, 2.7 µm). As a mobile phase, 0.1% orthophosphoric acid (mobile phase A) and 100% methanol (mobile phase B) were used. Other conditions included an injected volume of 10 µl and a flow rate of 1 mL/min. The following gradient was used for separation: 0 min 5% B, 3 min 30% B, 15 min 35% B, 22 min 37% B, 30 min 41% B, 32 min 45% B, 40 min 49% B, 45 min 80% B, 48 min 80% B, 50 min 5% B, 53 min 5% B. A stock solution for cyanidin 3-glucoside was prepared in 0.1% HCl in methanol, while other polyphenol standards were prepared in 100% methanol. The calibration curves of the polyphenol standards were constructed at different concentrations for cyanidin 3-glucoside, ellagic acid, gallic acid, caffeic acid, chlorogenic acid, p-coumaric acid, rutin, quercetin and hesperidin. UV/Vis spectra were recorded in the range of 190 to 600 nm. The identification was performed by comparing retention times and the UV/Vis spectrum of peaks in extracts with those of the standards. In addition, to confirm the identification, extracts were spiked with standards. Cyanidin 3-dioxalylglucoside was tentatively identified according to the literature [35,50], and quantified using cyanidin 3-glucoside.

### 3.7. Determination of Antioxidant Activity

Four spectrophotometric methods (DPPH, ABTS, FRAP and CUPRAC) were used for antioxidant activity determination. The DPPH method was performed according to Brand-Williams et al. [51], with some modifications. In short, 3 mL of DPPH solution (0.5 mM) was transferred to a glass tube containing 0.2 mL of sample. The absorbance was measured at 517 nm after 15 min in the dark. For the ABTS method according to Arnao et al. [52], with some modifications, 0.2 mL of the sample was mixed with 3 mL of ABTS reagent. After 95 min in the dark, the absorbance was measured at 734 nm. Furthermore, for determination of ferric reducing ability [53], 0.2 mL of sample was mixed with 3 mL of FRAP reagent and left in the dark for 30 min. The absorbance was then measured at 593 nm. Cupricreducing antioxidant capacity (CUPRAC) was performed as follows: 1 mL of CuCl_2_ (10 mM), neocuproine (7.5 mM) and ammonium acetate buffer (1 M, pH 7.0) were added to a glass tube; the sample and distilled water were added, to a total volume of 1.1 mL; after 30 min left in the dark, the absorbance at 450 nm was read [54]. Results were expressed as micromoles of Trolox equivalent per 100 g of sample (µmol TE/100 g).

### 3.8. Fourier Transform Infrared with Attenuated Total Reflection (FTIR-ATR) Spectroscopy Analysis

The FTIR-ATR (Cary 630, Agilent, Santa Clara, CA, USA) technique was used for obtaining the infrared (IR) spectra of complexes. The observed IR spectra are analyzed as the absorbance of different complexes versus the wavenumber range from 4000 cm^−1^ to 600 cm^−1^.

### 3.9. Statistical Analysis

Statistical analyses were conducted using the software program STATISTICA 13.1 (StatSoft Inc., Tulsa, OK, USA). Data were evaluated using an analysis of variance (ANOVA) and Fisher’s least significant difference (LSD) test, with the significance defined at *p* < 0.05. All results were expressed as the mean values ± standard deviation. Spectrophotometric assays were performed in triplicate, while HPLC and FTIR were performed in duplicate.

## 4. Conclusions

The results presented in this study indicate the possibility of using citrus fiber as a delivery system for blackberry juice polyphenols. The encapsulation of such compounds is a critical step in the development of efficient delivery systems, because the interactions between carriers and the loaded molecules can have an impact on the encapsulated system (storage stability of encapsulates, retention of polyphenols, changes in chemical structure of polyphenols). It can be concluded that the adsorption of polyphenols largely depends on the amount of fiber, and the complex with 1% fiber had the highest concentration of polyphenols and antioxidant activity. These complexes, rich in hesperidin from citrus and adsorbed polyphenols from blackberry juice, could be used to develop and/or improve novel, functional foods with various health-promoting properties. Future studies should examine the actual impact of these potential ingredients on product quality and stability, in order to enable technological applications in the food industry.

## Figures and Tables

**Table 1:** Measured values of a_w_ in complexes after preparation, and after storage.

CF_1%/B	CF_2%/B	CF_4%/B
After preparation		
0.38	0.34	0.32
After storage		
0.45	0.40	0.40

a_w_–water activity; CF—citrus fiber; 1%, 2% and 3%—amounts of CF; B—blackberry juice.

**Table 2:** Characteristics of blackberry juice.

TPC (mg GAE/L)	647.4 ± 10.3
PC (mg PB2E/L)	6.37 ± 0.81
DPPH (µmol TE/100 mL)	2.39 ± 0.05
ABTS (µmol TE/100 mL)	5.55 ± 0.08
FRAP (µmol TE/100 mL)	0.57 ± 0.02
CUPRAC (µmol TE/100 mL)	30.30 ± 0.31
Individual polyphenols (mg/mL)	
Cyanidin 3-glucoside	573.86 ± 3.02
Cyanidin 3-dioxalylglucoside	212.76 ± 0.60
Ellagic acid	40.80 ± 0.11
p-Coumaric acid	41.10 ± 0.00
Caffeic acid	3.85 ± 0.04
Chlorogenic acid	31.50 ± 0.01
Gallic acid	47.35 ± 0.30
Quercetin	24.05 ± 0.02
Rutin	4.95 ± 0.00

TPC—total polyphenol content; PC—proanthocyanidin content; DPPH (2,2-diphenyl-1-picrylhydrazyl) - free radical scavenging activity; ABTS (2,2′-azino-bis(3-ethylbenzothiazoline-6-sulfonic acid) - radical scavenging activity; FRAP–ferric reducing antioxidant power; CUPRAC–cupric reducing antioxidant capacity; GAE, PB2E and TE—gallic acid, procyanidin B2 and trolox equivalents.

**Table 3:** Total polyphenols and proanthocyanidins of citrus fiber/blackberry juice complexes.

Samples	TPC (mg GAE/g)	PC (mg PB2E/g)
	After preparation	
CF_1%/B	12.89 ± 0.10 ^c^	1.90 ± 0.04 ^c^
CF_2%/B	7.49 ± 0.05 ^b^	1.54 ± 0.03 ^b^
CF_4%/B	6.27 ± 0.01 ^a^	1.15 ± 0.02 ^a^
	After storage	
CF_1%/B	6.47 ± 0.17 ^b^	1.42 ± 0.00 ^c^
CF_2%/B	5.24 ± 0.11 ^a^	1.13 ± 0.02 ^b^
CF_4%/B	4.96 ± 0.02 ^a^	0.93 ± 0.02 ^a^

TPC–total polyphenols content; PC–proanthocyanidins content; CF—citrus fiber; 1%, 2% and 3%—amounts of CF; B—blackberry juice; GAE and PB2E—gallic acid and procyanidin B2 equivalents. Within each column (separate for freshly prepared and stored samples), means followed by different letters of superscripts are significantly different at *p* ≤ 0.05 (ANOVA, Fisher’s least significant difference (LSD) test).

**Table 4:** Concentrations of individual polyphenols in citrus fiber/blackberry juice complexes (mg/100 g) after preparation, and after storage.

Polyphenols	CF_1%/B	CF_2%/B	CF_4%/B
After preparation			
Cyanidin 3-glucoside	246.45 ± 2.33 ^b^	243.57 ± 5.76 ^b^	160.78 ± 0.30 ^a^
Cyanidin 3-dioxalylglucoside	31.45 ± 0.04 ^c^	22.67 ± 0.04 ^b^	20.51 ± 0.02 ^a^
Ellagic acid	50.11 ± 0.47 ^c^	32.23 ± 1.27 ^b^	22.96 ± 0.08 ^a^
Quercetin	33.77 ± 0.33 ^c^	22.59 ± 0.15 ^b^	20.43 ± 0.12 ^a^
Hesperidin	1571.65 ± 4.33 ^c^	1422.25 ± 3.67 ^b^	1371.88 ± 9.38 ^a^
After storage			
Cyanidin 3-glucoside	213.36 ± 0.22 ^b^	215.12 ± 2.40 ^b^	138.32 ± 2.35 ^a^
Cyanidin 3-dioxalylglucoside	25.07 ± 0.20 ^b^	26.18 ± 0.06 ^c^	22.19 ± 0.08 ^a^
Ellagic acid	44.55 ± 0.55 ^c^	32.78 ± 0.39 ^b^	21.94 ± 0.73 ^a^
Quercetin	24.75 ± 0.02 ^c^	22.37 ± 0.07 ^b^	20.11 ± 0.05 ^a^
Hesperidin	1358.84 ± 4.29 ^b^	1379.66 ± 15.13 ^b^	1317.59 ± 12.79 ^a^

CF—citrus fiber; 1%, 2% and 3%—amounts of CF; B—blackberry juice. Within the rows, means followed by different superscript letters are significantly different at *p* ≤ 0.05 (ANOVA, Fisher’s LSD).

**Table 5:** Antioxidant activity of citrus fiber/blackberry juice complexes (µmol TE/100 g).

Samples	DPPH	ABTS	FRAP	CUPRAC
After preparation				
CF_1%/B	50.39 ± 1.54 ^b^	52.83 ± 0.03 ^c^	9.71 ± 0.51 ^b^	515.00 ± 10.00 ^b^
CF_2%/B	47.86 ± 1.96 ^b^	45.35 ± 0.08 ^b^	8.89 ± 0.60 ^b^	509.00 ± 12.00 ^b^
CF_4%/B	36.48 ± 1.77 ^a^	29.60 ± 0.14 ^a^	6.65 ± 0.36 ^a^	362.00 ± 4.00 ^a^
		After storage		
CF_1%/B	49.84 ± 1.13 ^c^	51.30 ± 0.03 ^c^	9.13 ± 0.47 ^c^	509.41 ± 2.57 ^c^
CF_2%/B	45.07 ± 0.71 ^b^	37.58 ± 0.74 ^b^	8.02 ± 0.10 ^b^	453.81 ± 1.50 ^b^
CF_4%/B	35.48 ± 0.38 ^a^	24.78 ± 0.47 ^a^	6.30 ± 0.19 ^a^	351.34 ± 2.79 ^a^

DPPH (2,2-diphenyl-1-picrylhydrazyl)—free radical scavenging activity; ABTS (2,2′-azino-bis(3-ethylbenzothiazoline-6-sulfonic acid)—radical scavenging activity; FRAP–ferric reducing antioxidant power; CUPRAC–cupric reducing antioxidant capacity; CF—citrus fiber; 1%, 2% and 3%—amounts of CF; B—blackberry juice; TE—trolox equivalent. Within each column (separate for freshly prepared and stored samples), means followed by different letters of superscripts are significantly different at *p* ≤ 0.05 (ANOVA, Fisher’s LSD).

## Data Availability

Not available.

## Sample Availability

Samples of the compounds are available from the authors.
